# Extended spiking neural P systems with white hole rules and their red–green variants

**DOI:** 10.1007/s11047-017-9649-7

**Published:** 2017-11-13

**Authors:** Artiom Alhazov, Rudolf Freund, Sergiu Ivanov, Marion Oswald, Sergey Verlan

**Affiliations:** 1 Institute of Mathematics and Computer Science, Academy of Sciences of Moldova, Str. Academiei 5, 2028 Chişinău, Moldova; 2 Faculty of Informatics, TU Wien, Favoritenstraße 9-11, 1040 Vienna, Austria; 3 Université Paris Est Créteil, Créteil, France

**Keywords:** Going beyond Turing, Red-green automata, Spiking neural P systems, White hole rules

## Abstract

We consider extended spiking neural P systems with the additional possibility of so-called “white hole rules”, which send the complete contents of a neuron to other neurons, and we prove that this extension of the original model can easily simulate register machines. Based on this proof, we then define red–green variants of these extended spiking neural P systems with white hole rules and show how to go beyond Turing with these red–green systems. We also discuss the number of actor neurons needed, and the relation of this model to some special variants of Lindenmayer systems.

## Introduction

Based on the biological background of neurons sending electrical impulses along axons to other neurons, several models were developed in the area of neural computation, e.g., see Maass (2002), Maass and Bishop (1999), and Gerstner and Kistler (2002). In the area of P systems, the model of *spiking neural P systems* was introduced in Ionescu et al. (2006). Whereas the basic model of membrane systems, see Păun (2000), reflects hierarchical membrane structures, the model of tissue P systems considers cells to be placed in the nodes of a graph. This variant was first considered in Păun et al. (2006) and then further elaborated, for example, in Freund et al. (2004) and Martín-Vide et al. (2002). In spiking neural P systems, the cells are arranged as in tissue P systems, but the contents of a cell (neuron) consists of a number of so-called *spikes*, i.e., of a multiset over a single object. The rules assigned to a neuron allow us to send information to other neurons in the form of electrical impulses (also called spikes) which are summed up at the target neuron; the application of the rules depends on the contents of the neuron and in the general case is described by regular sets. As inspired from biology, the neuron sending out spikes may be “closed” for a specific time period corresponding to the refraction period of a neuron; during this refraction period, the neuron is closed for new input and cannot get excited (“fire”) for spiking again.

The length of the axon may cause a time delay before a spike arrives at the target. Moreover, the spikes coming along different axons may cause effects of different magnitude. All these biologically motivated features were included in the model of extended spiking neural P systems considered in Alhazov et al. (2006), the most important theoretical feature being that neurons can send spikes along the axons with different magnitudes at different moments of time. In Wang et al. (2010), spiking neural P systems with weights on the axons and firing threshold were investigated, where the values of these weights and firing thresholds as well as the potential consumed by each rule could be natural numbers, integer numbers, rational numbers, and even (computable) real numbers.

In this paper, we will further extend the model of extended spiking neural P systems by using so-called “white hole rules”, which allow us to use the whole contents of a neuron and send it to other neurons, yet eventually multiplied by some constant rational number.

In the literature, several variants how to obtain results from the computations of a spiking neural P system have been investigated. For example, in Ionescu et al. (2006) the output of a spiking neural P system was considered to be the time between two spikes in a designated output neuron. It was shown how spiking neural P systems in that way can generate any recursively enumerable set of natural numbers. Moreover, a characterization of semilinear sets was obtained by spiking neural P system with a bounded number of spikes in the neurons. These results can also be obtained with even more restricted forms of spiking neural P systems, e.g., no time delay (refraction period) is needed, as it was shown in Ibarra et al. (2006). In Chen et al. (2006), the generation of strings (over the binary alphabet 0 and 1) by spiking neural P systems was investigated; due to the restrictions of the original model of spiking neural P systems, even specific finite languages cannot be generated, but on the other hand, regular languages can be represented as inverse-morphic images of languages generated by finite spiking neural P systems, and even recursively enumerable languages can be characterized as projections of inverse-morphic images of languages generated by spiking neural P systems. The problems occurring in the proofs are also caused by the quite restricted way the output is obtained from the output neuron as sequence of symbols 0 and 1. The strings of a regular or recursively enumerable language could be obtained directly by collecting the spikes sent by specific output neurons for each symbol.

In the extended model considered in Alhazov et al. (2006), a specific output neuron was used for each symbol. Computational completeness could be obtained by simulating register machines as in the proofs elaborated in the papers mentioned above, yet in an easier way using only a bounded number of neurons. Moreover, regular languages could be characterized by finite extended spiking neural P systems; again, only a bounded number of neurons was needed.

In this paper, we now extend this model of extended spiking neural P systems by also using so-called “white hole rules”, which may send the whole contents of a neuron along its axons, eventually even multiplied by a (positive) rational number. In that way, the whole contents of a neuron can be multiplied by a rational number, in fact, multiplied with or divided by a natural number. Hence, even one single neuron is able to simulate the computations of an arbitrary register machine.

The idea of consuming the whole contents of a neuron by white hole rules is closely related to the concept of the exhaustive use of rules, i.e., an enabled rule is applied in the maximal way possible in one step; P systems with the exhaustive use of rules can be used in the usual maximally parallel way on the level of the whole system or in the sequential way, for example, see Zhang et al. (2008, 2012). Yet all the approaches of spiking neural P systems with the exhaustive use of rules are mainly based on the classic definitions of spiking neural P systems, whereas the spiking neural P systems with white hole rules as investigated in Alhazov et al. (2015a) are based on the extended model as introduced in Alhazov et al. (2006). In this paper we now use this new model of spiking neural P systems with white hole rules together the idea of considering infinite computations on finite inputs, which will allow us to “go beyond Turing”.

Variants of how to “go beyond Turing” are discussed in van Leeuwen and Wiedermann (2012), for example, the definitions and results for red–green Turing machines can be found there. In Aman et al. (2014) the notion of red–green automata for register machines with input strings given on an input tape (often also called *counter automata*) was introduced and the concept of *red–green P automata* for several specific models of membrane systems was explained. Via red–green counter automata, the results for acceptance and recognizability of finite strings by red–green Turing machines were carried over to red–green P automata. The basic idea of red–green automata is to distinguish between two different sets of states (red and green states) and to consider infinite runs of the automaton on finite input objects (strings, multisets); allowed to change between red and green states more than once, red–green automata can recognize more than the recursively enumerable sets (of strings, multisets), i.e., in that way we can “go beyond Turing”. In the area of P systems, first attempts to do that can be found in Calude and Păun (2004) and Sosík and Valík (2006). Computations with infinite words by P automata were investigated in Freund et al. (2004).

The rest of the paper is organized as follows: In the next section, we recall some preliminary notions and definitions from formal language theory, especially the definition and some well-known results for register machines. Then we define red–green Turing machines and red–green register machines and recall some results from Aman et al. (2014). In Sect. 4 we recall the definitions of the extended model of spiking neural P systems as considered in Alhazov et al. (2006) as well as the most important results established there. Moreover, we show that extended spiking neural P systems with only one actor neuron have exactly the same computational power as register machines with only one register that can be decremented.

In Sect. 5, we define the model of extended spiking neural P systems extended by the use of white hole rules as introduced in Alhazov et al. (2015a). Besides giving some examples, for instance showing how Lindenmayer systems can be simulated by extended spiking neural P systems only using white hole rules, we prove that the computations of an arbitrary register machine can be simulated by only one single neuron equipped with the most powerful variant of white hole rules, i.e., extended spiking neural P systems equipped with white hole rules are even more powerful than extended spiking neural P systems, which need (at least) two neurons to be able to simulate the computations of an arbitrary register machine. Based on this result, we define the *red–green* variant of spiking neural P systems with white hole rules and show that their computational power is similar to the computational power of red–green register machines. A short summary of the results we obtained concludes the paper.

## Preliminaries

In this section we recall the basic elements of formal language theory and especially the definitions and results for register machines; we here mainly follow the corresponding section from Alhazov et al. (2006, 2015a).

For the basic elements of formal language theory needed in the following, we refer to any monograph in this area, in particular, to Rozenberg and Salomaa (1997). We just list a few notions and notations: $V^*$ is the free monoid generated by the alphabet *V* under the operation of concatenation and the empty string, denoted by $\lambda$, as unit element; for any $w\in V^*$, $\left| w\right|$ denotes the number of symbols in *w* (the *length* of *w*). ${\mathbb{N}}_+$ denotes the set of positive integers (natural numbers), ${\mathbb{N}}$ is the set of non-negative integers, i.e., ${\mathbb{N}}={\mathbb{N}}_+\cup \left\{ 0\right\}$, and $\mathbb{Z}$ is the set of integers, i.e., $\mathbb{Z}={\mathbb{N}}_+\cup \left\{ 0\right\} \cup -{\mathbb{N}}_+$. The interval of non-negative integers between *k* and *m* is denoted by $\left[ k..m\right]$, and $k\cdot {\mathbb{N}}_+$ denotes the set of positive multiples of *k*. Observe that there is a one-to-one correspondence between a set $M\subseteq {\mathbb{N}}$ and the one-letter language $L\left( M\right) =\left\{ a^n\mid n\in M\right\}$; e.g., *M* is a regular (semilinear) set of non-negative integers if and only if $L\left( M\right)$ is a regular language. By $FIN\left( {\mathbb{N}}^k\right)$, $REG\left( {\mathbb{N}}^k\right)$, and $RE\left( {\mathbb{N}}^k\right)$, for any $k\in {\mathbb{N}}$, we denote the sets of subsets of ${\mathbb{N}}^k$ that are finite, regular, and recursively enumerable, respectively.

By *REG* ($REG\left( V\right)$) and *RE* ($RE\left( V\right)$) we denote the family of regular and recursively enumerable languages (over the alphabet *V*, respectively). By $\varPsi _{T}\left( L\right)$ we denote the Parikh image of the language $L\subseteq T^*$, and by *PsFL* we denote the set of Parikh images of languages from a given family *FL*. In that sense, $PsRE\left( V\right)$ for a *k*-letter alphabet *V* corresponds with the family of recursively enumerable sets of *k*-dimensional vectors of non-negative integers.

### Register machines

The proofs of the results establishing computational completeness in the area of P systems often are based on the simulation of register machines; we refer to Minsky (1967) for original definitions, and to Freund and Oswald (2002) for the definitions we use in this paper:

An *n*-*register machine* is a tuple $M=\left( n,B,l_0,l_h,P\right)$, where *n* is the number of registers, *B* is a set of labels, $l_0\in B$ is the initial label, $l_h\in B$ is the final label, and *P* is the set of instructions bijectively labeled by elements of *B*. The instructions of *M* can be of the following forms:

- $$l_1:\left( ADD\left( r\right) ,l_2,l_3\right)$$, with $$l_1\in B\setminus \left\{ l_h\right\}$$, $$l_2,l_3\in B$$, $$1\le j\le n$$.
  Increases the value of register *r* by one, followed by a non-deterministic jump to instruction $l_2$ or $l_3$. This instruction is usually called *increment*.
- $$l_1:\left( SUB\left( r\right) ,l_2,l_3\right)$$, with $$l_1\in B\setminus \left\{ l_h\right\}$$, $$l_2,l_3\in B$$, $$1\le j\le n$$.
  If the value of register *r* is zero then jump to instruction $l_3$; otherwise, the value of register *r* is decreased by one, followed by a jump to instruction $l_2$. The two cases of this instruction are usually called *zero-test* and *decrement*, respectively.
- $$l_h:halt$$   (HALT instruction)
  Stop the machine. The final label $l_h$ is only assigned to this instruction.

A (non-deterministic) register machine *M* is said to generate a vector $\left( s_1,\ldots ,s_\beta \right)$ of natural numbers if, starting with the instruction with label $l_0$ and all registers containing the number 0, the machine stops (it reaches the instruction $l_h:halt$) with the first $\beta$ registers containing the numbers $s_1,\ldots ,s_\beta$ (and all other registers being empty).

Without loss of generality, in the succeeding proofs we will assume that in each ADD instruction $l_1:\left( ADD\left( r\right) ,l_2,l_3\right)$ and in each SUB instruction $l_1:\left( SUB\left( r\right) ,l_2,l_3\right)$ the labels $l_1,l_2,l_3$ are mutually distinct (for a short proof see Freund et al. 2004).

The register machines are known to be computationally complete, equal in power to (non-deterministic) Turing machines: they generate exactly the sets of vectors of non-negative integers which can be generated by Turing machines, i.e., the family *PsRE*.

Based on the results established in Minsky (1967), the results proved in Freund and Oswald (2002) and Freund and Păun (2004) immediately lead to the following result:

#### **Proposition 1**

*For any recursively enumerable set* $L\subseteq {\mathbb{N}}^\beta$ *of vectors of non-negative integers there exists a non-deterministic* $\left( \beta +2\right)$ *-register machine* *M* *generating* *L* *in such a way that, when starting with all registers* 1 *to* $\beta +2$ *being empty*, *M* *non-deterministically computes and halts with* $n_i$ *in registers* *i*, $1\le i\le \beta$, *and registers* $\beta +1$ *and* $\beta +2$ *being empty if and only if* $\left( n_1,\ldots ,n_\beta \right) \in L$. *Moreover, the registers* 1 *to* $\beta$ *are never decremented.*

When considering the generation of languages, we can use the model of a *register machine with output tape*, which also uses a tape operation:

- $$l_1:\left( write\left( a\right) ,l_2\right)$$
  Write symbol *a* on the output tape and go to instruction $l_2.$

We then also specify the output alphabet *T* in the description of the register machine with output tape, i.e., we write $M=\left( m,B,l_0,l_h,P,T\right)$.

The following result is folklore, too, e.g., see Minsky (1967):

#### **Proposition 2**

*Let* $L\subseteq T^*$ *be a recursively enumerable language. Then* *L* *can be generated by a register machine with output tape with* 2 *registers. Moreover, at the beginning and at the end of a successful computation generating a string* $w\in L$ *both registers are empty, and finally, only successful computations halt.*

### The arithmetical hierarchy

The Arithmetical Hierarchy—e.g., see Budnik (2006)—is usually developed with the universal ($\forall$) and existential ($\exists$) quantifiers restricted to the integers. Levels in the Arithmetical Hierarchy are labeled as $\varSigma _n$ if they can be defined by expressions beginning with a sequence of *n* alternating quantifiers starting with $\exists$; levels are labeled as $\varPi _n$ if they can be defined by such expressions of *n* alternating quantifiers that start with $\forall$. $\varSigma _0$ and $\varPi _0$ are defined as having no quantifiers and are equivalent. $\varSigma _1$ and $\varPi _1$ only have the single quantifier $\exists$ and $\forall$, respectively. We only need to consider alternating pairs of the quantifiers $\forall$ and $\exists$ because two quantifiers of the same type occurring together are equivalent to a single quantifier.

## Red–green automata

The exposition of this section mainly follows the corresponding section in Alhazov et al. (2015a).

In general, a red–green automaton *M* is an automaton whose set of internal states *Q* is partitioned into two subsets, $Q_{red}$ and $Q_{green}$, and *M* operates without halting. $Q_{red}$ is called the set of “red states”, $Q_{green}$ the set of “green states”. Moreover, we shall assume *M* to be deterministic, i.e., for each configuration there exists exactly one transition to the next one.

### Red–green turing machines

Red–green Turing machines, see van Leeuwen and Wiedermann (2012), can be seen as a type of $\omega$-Turing machines on finite inputs with a recognition criterion based on some property of the set(s) of states visited (in)finitely often, in the tradition of $\omega$-automata, for example, see Freund et al. (2004), i.e., we call an infinite run of the Turing machine *M* on input *w* *recognizing* if and only if

- no red state is visited infinitely often and
- some green states (one or more) are visited infinitely often.

A set of strings $L\subset \varSigma ^*$ is said to be *accepted* by *M* if and only if the following two conditions are satisfied:

1. $$L=\left\{ w\mid w\, \text{is recognized by}\, M\right\}$$.
2. For every string $w\notin L$, the computation of *M* on input *w* eventually stabilizes in red; in this case *w* is said to be *rejected*.

The phrase “mind change” is used in the sense of changing the color, i.e., changing from red to green or vice versa.

The following results were established in van Leeuwen and Wiedermann (2012):

#### **Theorem 1**

*A set of strings* *L* *is recognized by a red–green Turing machine with one mind change if and only if* $L\in \varSigma _1$, *i.e., if* *L* *is recursively enumerable.*

#### **Theorem 2**

(Computational power of red–green Turing machines)

1. *Red–green Turing machines recognize exactly the* $\varSigma _2$ *-sets of the Arithmetical Hierarchy.*
2. *Red–green Turing machines accept exactly those sets which simultaneously are* $\varSigma _2$- *and* $\varPi _2$-*sets of the Arithmetical Hierarchy.*

### Red–green register machines

In Aman et al. (2014), similar results as for red–green Turing machines were shown for red–green counter automata and register machines, respectively.

As it is well-known folklore, e.g., see Minsky (1967), the computations of a Turing machine can be simulated by a counter automaton with (only two) counters; in this paper, we will rather speak of a register machine with (two) registers and with string input. As for red–green Turing machines, we can also color the “states”, i.e., the labels, of a register machine $M=\left( m,B,l_0,l_h,P,T_{in}\right)$ by the two colors red and green, i.e., partition its set of labels *B* into two disjoint sets $B_{red}$ (red “states”) and $B_{green}$ (green “states”), and we then write $RM=\left( m,B,B_{red},B_{green},l_0,P,T_{in}\right)$, as we can omit the halting label $l_h$.

The following two lemmas were proved in Aman et al. (2014); the step from red–green Turing machines to red–green register machines is important for the succeeding sections, as usually register machines are simulated when proving a model of P systems to be computationally complete. Therefore, in the following we always have in mind this specific relation between red–green Turing machines and red–green register machines when investigating the infinite behavior of specific models of P automata, as we will only have to argue how red–green register machines can be simulated.

#### **Lemma 1**

*The computations of a red–green Turing machine* *TM* *can be simulated by a red–green register machine* *RM* *with two registers and with string input in such a way that during the simulation of a transition of* *TM* *leading from a state* *p* *with color* *c* *to a state* $p'$ *with color* $c'$ *the simulating register machine uses instructions with labels (“states”) of color* *c* *and only in the last step of the simulation changes to a label (“state”) of color* $c'$.

#### **Lemma 2**

*The computations of a red–green register machine* *RM* *with an arbitrary number of registers and with string input can be simulated by a red–green Turing machine* *TM* *in such a way that during the simulation of a computation step of* *RM* *leading from an instruction with label (“state”)* *p* *with color* *c* *to an instruction with label (“state”)* $p'$ *with color* $c'$ *the simulating Turing machine stays in states of color* *c* *and only in the last step of the simulation changes to a state of color* $c'$.

As an immediate consequence, the preceding two lemmas yield the characterization of $\varSigma _2$ and $\varSigma _2\cap \varPi _2$ by red–green register machines as Theorem 2 does for red–green Turing machines, see van Leeuwen and Wiedermann (2012):

#### **Theorem 3**

(Computational power of red–green register machines)

1. *A set of strings* *L* *is recognized by a red–green register machine with one mind change if and only if* $L\in \varSigma _1$, *i.e., if* *L* *is recursively enumerable.*
2. *Red–green register machines recognize exactly the* $\varSigma _2$-*sets of the Arithmetical Hierarchy.*
3. *Red–green register machines accept exactly those sets which simultaneously are* $\varSigma _2$-*and* $\varPi _2$-*sets of the Arithmetical Hierarchy.*

## Extended spiking neural P systems

The reader is supposed to be familiar with basic elements of membrane computing, e.g., from Păun (2002) and Păun et al. (2010); comprehensive information can be found on the P systems web page (www.ppage.psystems.eu). Moreover, for the motivation and the biological background of spiking neural P systems we refer the reader to Ionescu et al. (2006). The definition of an *extended spiking neural P system* is mainly taken from Alhazov et al. (2006), with the number of spikes *k* still be given in the “classical” way as $a^k$; later on, we simple will use the number *k* itself only instead of $a^k$.

The definitions given in the following are taken from Alhazov et al. (2006).

### **Definition 1**

An *extended spiking neural P system* (of degree $m\ge 1$) (an *ESNP system* for short) is a construct $\varPi =\left( m,S,R\right)$ where

- *m* is the number of *cells* (or *neurons*); the neurons are uniquely identified by a number between 1 and *m* (obviously, we could instead use an alphabet with *m* symbols to identify the neurons);
- *S* describes the *initial configuration* by assigning an initial value (of spikes) to each neuron; for the sake of simplicity, we assume that at the beginning of a computation we have no pending packages along the axons between the neurons;
- $R$ is a finite set of *rules* of the form $\left( i,E/a^k\rightarrow P;d\right)$ such that $i\in \left[ 1..m\right]$ (specifying that this rule is assigned to neuron *i*), $E\subseteq REG\left( \left\{ a\right\} \right)$ is the *checking set* (the current number of spikes in the neuron has to be from *E* if this rule shall be executed), $k\in {\mathbb{N}}$ is the “number of spikes” (the energy) consumed by this rule, *d* is the *delay* (the “refraction time” when neuron *i* performs this rule), and *P* is a (possibly empty) set of *productions* of the form $\left( l,w,t\right)$ where $l\in \left[ 1..m\right]$ (thus specifying the target neuron), $w\in \left\{ a\right\} ^*$ is the *weight* of the energy sent along the axon from neuron *i* to neuron *l*, and *t* is the time needed before the information sent from neuron *i* arrives at neuron *l* (i.e., the *delay along the axon*). If the checking sets in all rules are finite, then $\varPi$ is called a *finite ESNP system*.

### **Definition 2**

A *configuration* of the ESNP system is described as follows:

- for each neuron, the actual number of spikes in the neuron is specified;
- in each neuron *i*, we may find an “activated rule” $\left( i,E/a^k\rightarrow P;d'\right)$ waiting to be executed where $d'$ is the remaining time until the neuron spikes;
- in each axon to a neuron *l*, we may find pending packages of the form $\left( l,w,t'\right)$ where $t'$ is the remaining time until $\left| w\right|$ spikes have to be added to neuron *l* provided it is not closed for input at the time this package arrives.

A *transition* from one configuration to another one now works as follows:

- for each neuron *i*, we first check whether we find an “activated rule” $\left( i,E/a^k\rightarrow P;d'\right)$ waiting to be executed; if $d'=0$, then neuron *i* “spikes”, i.e., for every production $\left( l,w,t\right)$ occurring in the set *P* we put the corresponding package $\left( l,w,t\right)$ on the axon from neuron *i* to neuron *l*, and after that, we eliminate this “activated rule” $\left( i,E/a^k\rightarrow P;d'\right)$;
- for each neuron *l*, we now consider all packages $\left( l,w,t'\right)$ on axons leading to neuron *l*; provided the neuron is not closed, i.e., if it does not carry an activated rule $\left( i,E/a^k\rightarrow P;d'\right)$ with $d'>0$, we then sum up all weights *w* in such packages where $t'=0$ and add this sum of spikes to the corresponding number of spikes in neuron *l*; in any case, the packages with $t'=0$ are eliminated from the axons, whereas for all packages with $t'>0$, we decrement $t'$ by one;
- for each neuron *i*, we now again check whether we find an “activated rule” $\left( i,E/a^k\rightarrow P;d'\right)$ (with $d'>0$) or not; if we have not found an “activated rule”, we now may apply any rule $\left( i,E/a^k\rightarrow P;d\right)$ from *R* for which the current number of spikes in the neuron is in *E* and then put a copy of this rule as “activated rule” for this neuron into the description of the current configuration; on the other hand, if there still has been an “activated rule” $\left( i,E/a^k\rightarrow P;d'\right)$ in the neuron with $d'>0$, then we replace $d'$ by $d'-1$ and keep $\left( i,E/a^k\rightarrow P;d'-1\right)$ as the “activated rule” in neuron *i* in the description of the configuration for the next step of the computation.

After having executed all the substeps described above in the correct sequence, we obtain the description of the new configuration. A *computation* is a sequence of configurations starting with the initial configuration given by *S*. A computation is called *successful* if it halts, i.e., if no pending package can be found along any axon, no neuron contains an activated rule, and for no neuron, a rule can be activated.

In the original model introduced in Ionescu et al. (2006), in the productions $\left( l,w,t\right)$ of a rule $\left( i,E/a^k\rightarrow \left\{ \left( l,w,t\right) \right\} ;d\right)$, only $w=a$ (for *spiking rules*) or $w=\lambda$ (for *forgetting rules*) as well as $t=0$ was allowed (and for forgetting rules, the checking set *E* had to be finite and disjoint from all other sets *E* in rules assigned to neuron *i*). Moreover, reflexive axons, i.e., leading from neuron *i* to neuron *i*, were not allowed, hence, for $\left( l,w,t\right)$ being a production in a rule $\left( i,E/a^k\rightarrow P;d\right)$ for neuron *i*, *l* $\ne i$ was required. Yet the most important extension is that different rules for neuron *i* may affect different axons leaving from it whereas in the original model the structure of the axons (called synapses there) was fixed. In Alhazov et al. (2006), the sequence of substeps leading from one configuration to the next one together with the interpretation of the rules from *R* was taken in such a way that the original model can be interpreted in a consistent way within the extended model introduced in that paper. As mentioned in Alhazov et al. (2006), from a mathematical point of view, another interpretation would have been even more suitable: whenever a rule $\left( i,E/a^k\rightarrow P;d\right)$ is activated, the packages induced by the productions $\left( l,w,t\right)$ in the set *P* of a rule $\left( i,E/a^k\rightarrow P;d\right)$ activated in a computation step are immediately put on the axon from neuron *i* to neuron *l*, whereas the delay *d* only indicates the refraction time for neuron *i* itself, i.e., the time period this neuron will be closed. The delay *t* in productions $\left( l,w,t\right)$ can be used to replace the delay in the neurons themselves in many of the constructions elaborated, for example, in Ionescu et al. (2006), Păun et al. (2006), and Chen et al. (2006). Yet as in (the proofs of computational completeness given in) Alhazov et al. (2006), we shall not need any of the delay features in this paper, hence we need not go into the details of these variants of interpreting the delays.

Depending on the purpose the ESNP system is to be used, some more features have to be specified: for generating *k*-dimensional vectors of non-negative integers, we have to designate *k* neurons as *output neurons*; the other neurons then will also be called * actor neurons*. There are several possibilities to define how the output values are computed; according to Ionescu et al. (2006), we can take the distance between the first two spikes in an output neuron to define its value. As in Alhazov et al. (2006), also in this paper, we take the number of spikes at the end of a successful computation in the neuron as the output value. For generating strings, we do not interpret the spike train of a single output neuron as done, for example, in Chen et al. (2006), but instead consider the sequence of spikes in the output neurons each of them corresponding to a specific terminal symbol; if more than one output neuron spikes, we take any permutation of the corresponding symbols as the next substring of the string to be generated.

### *Remark 1*

As already mentioned, there is a one-to-one correspondence between (sets of) strings $a^k$ over the one-letter alphabet $\left\{ a\right\}$ and the corresponding non-negative integer *k*. Hence, in the following, we will consider the checking sets *E* of a rule $\left( i,E/a^k\rightarrow P;d\right)$ to be sets of non-negative integers and write *k* instead of $a^k$ for any $w=a^k$ in a production $\left( l,w,t\right)$ of *P*. Moreover, if no delays *d* or *t* are needed, we simply omit them. For example, instead of $\left( 2,\left\{ a^i\right\} /a^{i}\rightarrow \left\{ \left( 1,a,0\right) ,\left( 2,a^j,0\right) \right\} ;0\right)$ we write $\left( 2,\left\{ i\right\} /i\rightarrow \left\{ \left( 1,1\right) ,\left( 2,j\right) \right\} \right)$.

### ESNP systems as generating devices

As in Alhazov et al. (2006), we first consider extended spiking neural P systems as generating devices. The following example gives a characterization of regular sets of non-negative integers:

#### *Example 1*

Any semilinear set of non-negative integers *M* can be generated by a finite ESNP system with only two neurons.

Let *M* be a semilinear set of non-negative integers and consider a regular grammar *G* generating the language $L\left( G\right) \subseteq \left\{ a\right\} ^*$ with $N\left( L\left( G\right) \right) =M$; without loss of generality we assume the regular grammar to be of the form $G=\left( N,\left\{ a\right\} ,A_1,P\right)$ with the set of non-terminal symbols *N*, $N=\left\{ A_i\mid 1\le i\le m\right\}$, the start symbol $A_1$, and *P* the set of regular productions of the form $B\rightarrow aC$ with $B,C\in N$ and $A\rightarrow \lambda$. We now construct the finite ESNP system $\varPi =\left( 2,S,R\right)$ that generates an element of *M* by the number of spikes contained in the output neuron 1 at the end of a halting computation: we start with one spike in neuron 2 (representing the start symbol $A_1$ and no spike in the output neuron 1, i.e., $S=\left\{ \left( 1,0\right) ,\left( 2,1\right) \right\}$. The production $A_i\rightarrow aA_j$ is simulated by the rule $\left( 2,\left\{ i\right\} /i\rightarrow \left\{ \left( 1,1\right) ,\left( 2,j\right) \right\} \right)$ and $A_i\rightarrow \lambda$ is simulated by the rule $\left( 2,\left\{ i\right\} /i\rightarrow \emptyset \right)$, i.e., in sum we obtain

$$\begin{aligned} \varPi= & {} \left( 2,S,R\right) ,\\ S= & {} \left\{ \left( 1,0\right) ,\left( 2,1\right) \right\} ,\\ R= & {} \left\{ \left( 2,\left\{ i\right\} /i\rightarrow \left\{ \left( 1,1\right) ,\left( 2,j\right) \right\} \right) \right. \mid \\&\left. \ 1\le i,j\le m,\ A_i\rightarrow aA_j\in P\right\} \\&\cup \left\{ \left( 2,\left\{ i\right\} /i\rightarrow \emptyset \right) \mid 1\le i\le m,A_i\rightarrow \lambda \in P\right\} . \end{aligned}$$

Neuron 2 keeps track of the actual non-terminal symbol and stops the derivation as soon as it simulates a production $A_i\rightarrow \lambda$, because finally neuron 2 is empty. In order to guarantee that this is the only way how we can obtain a halting computation in $\varPi$, without loss of generality we assume *G* to be reduced, i.e., for every non-terminal symbol *A* from *N* there is a regular production with *A* on the left-hand side. These observations prove that we have $N\left( L\left( G\right) \right) =M$.

The following results were already proved in Alhazov et al. (2006):

#### **Lemma 3**

*For any ESNP system where during any computation only a bounded number of spikes occurs in the actor neurons, the generated language is regular.*

#### **Theorem 4**

*Any regular language* *L* *with* $L\subseteq T^*$ *for a terminal alphabet* *T* *with* $card\left( T\right) =n$ *can be generated by a finite ESNP system with* $n+1$ *neurons. On the other hand, every language generated by a finite ESNP system is regular.*

#### **Corollary 1**

*Any semilinear set of* *n*-*dimensional vectors can be generated by a finite ESNP system with* $n+1$ *neurons. On the other hand, every set of* *n*-*dimensional vectors generated by a finite ESNP system is semilinear.*

#### **Theorem 5**

*Any recursively enumerable language* *L* *with* $L\subseteq T^*$ *for a terminal alphabet* *T* *with* $card\left( T\right) =n$ *can be generated by an ESNP system with* $n+2$ *neurons.*

#### **Corollary 2**

*Any recursively enumerable set of* *n* *-dimensional vectors can be generated by an ESNP system with* $n+2$ *neurons.*

Besides these results already established in Alhazov et al. (2006), we now prove a characterization of languages and sets of (vectors of) natural numbers generated by ESNPS with only one neuron. Roughly speaking, having only one actor neuron corresponds with, besides output registers, having only one register which can be decremented.

#### **Lemma 4**

*For any ESNP system with only one actor neuron we can effectively construct a register machine with output tape and only one register that can be decremented, generating the same language, respectively a register machine with one register that can be decremented, generating the same set of (vectors of) natural numbers.*

#### *Proof*

First we notice that the delays would not matter: the overall system is sequential, and therefore it is always possible to pre-compute what happens until the actor neuron re-opens; the weight of all pending packages is also bounded. All the details of storing and managing all these features by the finite control of the register machines are tedious, but very much straightforward. In the following, we therefore assume that the ESNPS is given as:

$$\begin{aligned} \varPi= & {} (n+1,S,R),\\ S= & {} \{(1,m_1),\ldots ,(n,m_n),(n+1,m_{n+1})\},\\ R= & {} \{(n+1,E_r/i_r\rightarrow \{(1,p_{r,1}),\ldots ,(n,p_{r,n}),\\&\ (n+1,p_{r,n+1})\})\mid 1\le r\le q\}. \end{aligned}$$

Thus, given *n*, $\varPi$ can be specified by the following non-negative integers: the number *q* of rules, initial spikes $m_1,\ldots ,m_n,m_{n+1}$, and, for every rule *r*, the following ingredients: the number $i_r$ of consumed spikes, the numbers $p_{r,1},\ldots ,p_{r,n+1}$ of produced spikes, and the regular sets $E_r$ of numbers. Note that, as it will be obvious later, it is enough to only consider the case $m_1=\cdots =m_n=0$, because otherwise placing the initial spikes can be done by a 1-register machine in a preparatory phase, before switching to the instruction corresponding to starting the simulation.

The main challenge of the construction is to remember the actual “status” of the regular checking sets. It is known that every regular set *E* of numbers is semilinear, and it is possible to write $E_r=\bigcup _{j=1}^{l_r}(k_r{\mathbb{N}}+d_{r,j})\cup D_r$, i.e., all the linear sets constituting $E_r$ can be reduced to a common period $k_r$, and an additional finite set. Then, we can take a common multiple *k* of periods $k_r$, and represent each checking set as $E_r=\left( k{\mathbb{N}}_++\{d'_{r,j}\mid 1\le j\le l'_r\}\right) \cup D'_r$, where $D'_r$ is finite.

Finally, take a number *M* such that *M* is a multiple of *k*, that *M* is larger than any element of $D_r$, $1\le r\le q$, that *M* is larger than any number $d'_{r,j}$, $1\le j\le l'_r$, $1\le r\le q$, that *M* is larger than any of $i_r$ and $p_{r,n+1}$, $1\le r\le q$. Then, if neuron $n+1$ has *N* spikes, the following properties hold:

- rule *r* is applicable if and only if $N\in E_r$ in case when $i_r\le N<M$, and if and only if $M+(N\,{\mathrm{mod}}\,M)\in E_r$ in case when $N\ge M$,
- the difference between the number of spikes in neuron $n+1$ in two successive configurations is not larger than *M*.

For neuron $n+1$, $Mk+j$ spikes (where $0\le j\le M-1$) will be represented by value *k* of register 1 and state *j*.

We simulate $\varPi$ by a register machine *R* with one register and an output tape of *m* symbols. Before we proceed, we need to remark that, without restricting the generality, we may have an arbitrary set of “next instructions” instead of $\{l_2,l_3\}$ in $l_1:(ADD(r),l_2,l_3)$, and arbitrary sets of “next instructions” instead of $\{l_2\}$ and $\{l_3\}$ in $l_1:(SUB(r),l_2,l_3)$. Indeed, non-determinism between choice of multiple instructions can be implemented by an increment followed by a decrement in each case, as many times as needed for the corresponding set of “next instructions”. Clearly, $l_1:(ADD(r),\{l_2\})$ is just a shorter form of $l_1:(ADD(r),l_2,l_2)$.

Finally, besides instructions *ADD*(*r*), *SUB*(*r*), *write*(*a*) and *halt*, we introduce the notation of *NOP*, meaning only a switch to a different instruction without modifying the register. This will greatly simplify the construction below, and such a notation can be reduced to either compressing the rules (by substituting the instruction label with the label of the next instruction in all other instructions), or be simulated by an *ADD*(1) instruction, followed by a *SUB*(1) instruction.

We take $b(m_{n+1}\,{\mathrm{mod}}\,M)$ as the starting state of *R*, and the starting value of register 1 is $m_{n+1}{\mathrm{div}}\ M$.

For every class modulo *M*, $0\le j\le M-1$, we define sets

$$\begin{aligned} L_{j,0}= &\, {} \{l_{r,0}\mid 1\le r\le q,\ j\in E_r,\ i_r\ge j\},\\ L_{j,+}= &\, {} \{l_{r,+}\mid 1\le r\le q,\ j+M\in E_r\} \end{aligned}$$

of applicable rules corresponding to remainder *j*, subscripts 0 and $+$ represent cases of having less than *M* spikes, and at least *M* spikes, respectively. Let us redefine any of these sets to $\{l_h\}$ if the expression above is empty.

We proceed with the actual simulation. A rule

$$\begin{aligned} \left( n+1,E_r/i_r\rightarrow \left\{ (1,p_{r,1}),\ldots ,(n,p_{r,n}), (n+1,p_{r,n+1})\right\} \right) \end{aligned}$$

can be simulated by the following rules of *R*:

$$\begin{aligned}&b(j):(S(1),L_{j,+},L_{j,0}),\quad l_r\in L_{j,0};\\&\quad l_{r,\alpha }:\ldots , (\text{a sequence of } \,p_{r_1} \,\text{instructions }\\&\quad \ldots , write(a_1), \ldots , p_{r_n} \,\text{instructions} write(a_n),\\&\quad \ldots l'_{r,\alpha },\text{and} p_{r_{n+1}} \text{instructions } ADD(1)), \alpha \in \{0,+\};\\&\quad l'_{r,+}:(NOP,\{b((j-i_r+p_{r,n+1}){\mathrm{mod}}\,M)\}),\\&\quad \text{ if } j-i_r+p_{r,n+1}<0;\\&\quad l'_{r,+}:(ADD(1),\{l'_{r,0}\}), \text{ if } j-i_r+p_{r,n+1}<M;\\&\quad l'_{r,0}:(NOP,\{b((j-i_r+p_{r,n+1}){\mathrm{mod }}\, M)\}),\\&\quad \text{ if } j-i_r+p_{r,n+1}<M;\\&\quad l'_{r,0}:(ADD(1),\{b((j-i_r+p_{r,n+1}){\mathrm{mod}}\, M)\}),\\&\quad \text{ if } j-i_r+p_{r,n+1}\ge M;\\&\quad l_h:halt. \end{aligned}$$

Indeed, instruction *b*(*j*) corresponds to checking whether neuron $n+1$ has at least *M* spikes, transitioning into the halting instruction, or into the set of instructions associated with the corresponding applicable rules, in the context of the result of the checking mentioned above. Sending spikes to output neurons is simulated by writing the corresponding symbols on the tape. This goal is obtained, knowing values *j*, $i_r$, $p_{r,n+1}$, and whether neuron 1 had at least *M* spikes or not, by transitioning to instruction $b((j-i_r+p_{r,n+1}){\mathrm{mod}}\, M)$ after incrementing register 1 the needed number of times (0, 1 or 2), which is equal to $\left( j-i_r+p_{r,n+1}{\mathrm{div}}\ M\right) +d$, where $d=0$ if neuron 1 had at least *M* spikes, and $d=1$ otherwise (to compensate for the subtraction done by instruction *b*(*j*) in the initial checking). The simulation of instructions continues until we reach the situation where no rules of the underlying spiking system are applicable, transitioning to some $L_{j,\alpha }=\{l_h\}$.

Finally, let us formally describe the instruction sequences from $l_{r,\alpha }$ to $l'_{r,\alpha }$. For the sake of simplicity of notation, we do not mention subscripts $r,\alpha$ in the notation of the intermediate instructions, keeping in mind that these are different instructions for different $r,\alpha$. The difficulty for generating the string languages is that, by the definition, all permutations are to be considered if spikes are sent to multiple neurons $1,\ldots ,m$.

$$\begin{aligned}&l_{r,\alpha }:(NOP,\{s(p_{r_1},\ldots ,p_{r_n})\});\\&\quad s(i_1,\ldots ,i_n):\\&\quad \ (NOP,\{s^k(i_1,\ldots ,i_n) \mid i_k>0,\ 1\le k\le n\}),\\&\quad 0\le i_j\le p_{r_j},\ 1\le j\le n, (i_1,\ldots ,i_n)\ne (0,\ldots ,0);\\&\quad s^{(k)}(i_1,\ldots ,i_n):(write(a_k),\{s(i'_1,\ldots ,i'_n)\}),\\&\quad i'_k=i_k-1, \text{ and } i'_j=i_j,\quad 1\le j\le n,\ j\ne k,\\&\quad 0\le i_j\le p_{r_j},\ 1\le j\le n, (i_1,\ldots ,i_n)\ne (0,\ldots ,0);\\&\quad s(0,\ldots ,0):(NOP,\{t(p_{r_{n+1}})\});\\&\quad t(i):(ADD(n+1),t(i-1)),\quad 1\le i\le p_{r_{n+1}};\\&\quad t(0):(NOP,l'_{r,\alpha }). \end{aligned}$$

The rules above describe precisely the following behavior: to produce any sequence with the desired numbers of occurrences of symbols $a_1,\ldots ,a_n$, a symbol is non-deterministically chosen (out of those, the current desired number of occurrences of which is positive) and written, iterating until all desired symbols are written.

Next, the register is incremented the needed number of times. This finishes the explanation of the instruction sequences from $l_{r,\alpha }$ to $l'_{r,\alpha }$, as well as the explanation of the simulation.

Therefore, the class of languages generated by ESNP systems with only one neuron containing rules and *n* output neurons is included in the class of languages generated by 1-register machines with an output tape of *n* symbols.

Applying Parikh mapping to both classes, just replacing *write*-instructions by *ADD*-instructions on new registers associated with these symbols, it follows that the class of sets of vectors generated by ESNP systems with only one neuron containing rules and *n* output neurons is included in the class of sets of vectors generated by $n+1$-register machines where all registers except one are restricted to be increment-only. These observations conclude the proof. $\square$

The inclusions formulated at the end of the proof given above are actually characterizations, as we can also prove the opposite inclusion.

#### **Lemma 5**

*For any register machine with output tape with only one register that can be decremented respectively for any register machine with only one register that can be decremented we can effectively construct an ESNP system generating the same language respectively the same set of (vectors of) natural numbers.*

#### *Proof*

By definition, output registers can only be incremented, so the main computational power lies in the register which can also be decremented. The decrementable register can be simulated together with storing the actual state by storing the number $dn+c_i$ where: *n* is the actual contents of the register, $c_i$ is a number encoding the i-th instruction of the register machine, and *d* is a number bigger than all $c_i$. Then incrementing this first register by an instruction $c_i$ and jumping to $c_j$ means consuming $c_i$ and adding $d+c_j$ in the actor neuron, provided the checking set guarantees that the actual contents is an element of $d{\mathbb{N}}+c_i$. Decrementing means consuming $d+c_i$ and adding $c_j$ in the actor neuron, provided the checking set guarantees that the actual contents is an element of $d{\mathbb{N}}_++c_i$; if $n=0$, then $c_i$ is consumed and $c_k$ is added in the actor neuron with $c_k$ being the instruction to continue in the zero case. At the same time, with each of these simulation steps, the output neurons can be incremented in the exact way as the output registers; in the case of register machines with output tape, a spike is sent to the output neuron representing the symbol to be written. Further details of this construction are left to the reader. $\square$

## ESNP systems with white hole rules

In this section, we recall the definition of extended spiking neural P systems with white hole rules as introduced in Alhazov et al. (2015a). We will show that with this new variant of extended spiking neural P systems, computational completeness can already be obtained with only one actor neuron, by proving that the computations of any register machines can already be simulated in only one neuron equipped with the most general variant of white hole rules. Using this single actor neuron to also extract the final result of a computation, we even obtain weak universality with only one neuron.

As already mentioned in Remark 1, we are going to describe the checking sets and the number of spikes by non-negative integers. The following definition is an extension of Definition 1:

### **Definition 3**

An *extended spiking neural P system with white hole rules* (of degree $m\ge 1$) (in the following we shall simply speak of an *EESNP system*) is a construct $\varPi =\left( m,S,R\right)$ where

- *m* is the number of *cells *(or *neurons*); the neurons are uniquely identified by a number between 1 and *m*;
- *S* describes the *initial configuration* by assigning an initial value (of spikes) to each neuron;
- $R$ is a finite set of *rules* either being a *white hole rule* or a rule of the form as already described in Definition 3 $\left( i,E/k\rightarrow P;d\right)$ such that $i\in \left[ 1..m\right]$ (specifying that this rule is assigned to neuron *i*), $E\subseteq REG\left( {\mathbb{N}} \right)$ is the *checking set* (the current number of spikes in the neuron has to be from *E* if this rule shall be executed), $k\in {\mathbb{N}}$ is the “number of spikes” (the energy) consumed by this rule, *d* is the *delay* (the “refraction time” when neuron *i* performs this rule), and *P* is a (possibly empty) set of *productions* of the form $\left( l,w,t\right)$ where $l\in \left[ 1..m\right]$ (thus specifying the target neuron), $w\in {\mathbb{N}}$ is the *weight* of the energy sent along the axon from neuron *i* to neuron *l*, and *t* is the time needed before the information sent from neuron *i* arrives at neuron *l* (i.e., the *delay along the axon*). A *white hole rule* is of the form $\left( i,E/all \rightarrow P; d\right)$ where *all* means that the whole contents of the neuron is taken out of the neuron; in the productions $\left( l,w,t\right)$, either $w\in {\mathbb{N}}$ as before or else $w=\left( all +p\right) \cdot q+z$ with $p,q,z\in {\mathbb{Q}}$; provided $\left( c +p\right) \cdot q+z$, where *c* denotes the contents of the neuron, is non-negative, then $\left\lfloor \left( c +p\right) \cdot q+z\right\rfloor$ is the number of spikes put on the axon to neuron *l*.
  If the checking sets in all rules are finite, then $\varPi$ is called a *finite EESNP system*.

Allowing the white hole rules having productions being of the form $w=\left( all +p\right) \cdot q+z$ with $p,q,z\in \mathbb{Q}$ is a very general variant, which can be restricted in many ways, for example, by taking $z\in \mathbb{Z}$ or omitting any of the rational numbers $p,q,z\in \mathbb{Q}$ or demanding them to be in ${\mathbb{N}}$ etc.

Obviously, every ESNP system also is an EESNP system, but without white hole rules, and a finite EESNP system also is a finite ESNP system, as in this case the effect of white hole rules is also bounded, i.e., even with allowing the use of white hole rules, the following lemma as a counterpart of Lemma 3 is still valid:

### **Lemma 6**

*For any EESNP system where during any computation only a bounded number of spikes occurs in the actor neurons, the generated language is regular.*

Hence, in the following our main interest is in EESNP systems which really make use of the whole power of white hole rules.

EESNP systems can also be used for computing functions, not only for generating sets of (vectors of) integer numbers. As a simple example, we show how the function $n\mapsto 2^{n+1}$ can be computed by a deterministic EESPNS, which only has exactly one rule in each of its two neurons; the output neuron 2 in this case is not free of rules.

### *Example 2*

*Computing* $$n\mapsto 2^{n+1}$$ 
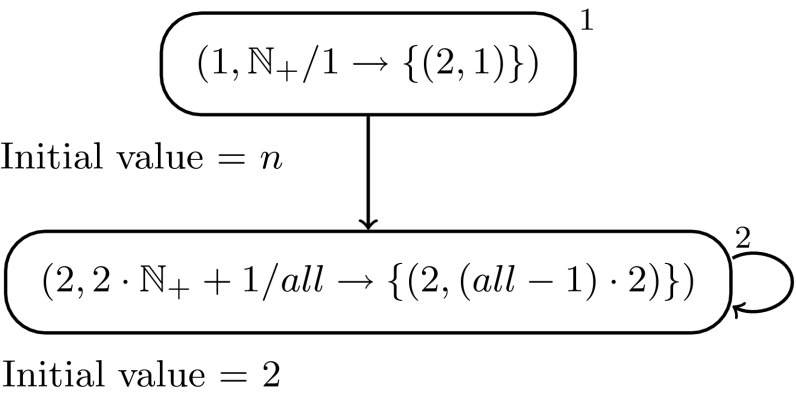


The rule $\left( 2,2\cdot {\mathbb{N}}_++1/all \rightarrow \left\{ \left( 2, \left( all -1\right) \cdot 2\right) \right\} \right)$ could also be written as $\left( 2,2\cdot {\mathbb{N}}_++1/all \rightarrow \left\{ \left( 2,\left( all\right) \cdot 2-2\right) \right\} \right)$. In both cases, starting with the input number *n* (of spikes) in neuron 1, with each decrement in neuron 1, the contents of neuron 2 (not taking into account the enabling spike from neuron 1) is doubled. The computation stops with $2^{n+1}$ in neuron 1, as with 0 in neuron 1 no enabling spike is sent to neuron 2 any more, hence, the firing condition is not fulfilled any more.

We remark that, if the initial value of neuron 2 is 1 (instead of 2), the function $n\mapsto 2^n$ will be computed (instead of $n\mapsto 2^{n+1}$). Indeed, if $n=0$, the system halts immediately and the value of the second neuron is $2^0=1$. If $n=1$, neuron 1 spikes once increasing the value of the second neuron to $2^1=2$, which is not enough for it to spike ($2\not \in 2\cdot {\mathbb{N}}_++1$), so the system halts. For values $n>1$, neuron 2 will start spiking at the second step of evolution, doubling its contents at each subsequent step; it will therefore contain $2^k$ spikes at the *k*-th evolution step.

Note that, when the initial value of neuron 2 is 2, the system satisfies the property that the second neuron spikes whenever the first one does. If we set the initial value of neuron 2 to 1, however, the second neuron never spikes before the first neuron spikes once.

### Pure white hole model

#### *Example 3*

Pure White Hole Model of EESNPS for DT0L Systems

Let $G=\left( \left\{ a\right\} ,P,a^s\right)$ be a Lindenmayer system with the axiom $a^s$ and the finite set of tables *P* each containing a finite set of parallel productions of the form $a\rightarrow a^k$. Such a system is called a tabled Lindenmayer system, abbreviated *T*0*L* system, and it is called deterministic, abbreviated *DT*0*L* system, if each table contains exactly one rule. Now let $G=\left( \left\{ a\right\} ,P,a^s\right)$ be a *DT*0*L* system with $P=\left\{ \left\{ a\rightarrow a^{k_i}\right\} \mid 1\le i\le n\right\}$. Then the following EESNPS using only white hole rules computes the same set of natural numbers as are represented by the language generated by *G*, with the results being taken with *unconditional halting*, i.e., taking a result at every moment, see Beyreder and Freund (2009). 
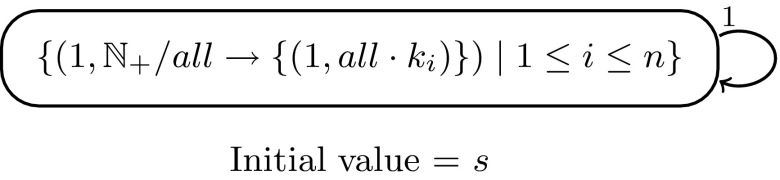


If we want to generate with normal halting, we have to add an additional output neuron 2 and an additional rule $\left\{ \left( 1,\mathbb{N_+}/all \rightarrow \left\{ \left( 2,all \cdot 1\right) \right\} \right) \right\}$ in neuron 1 which at the end moves the contents of neuron 1 to neuron 2.

To consider the generalization of the example above to multiple neurons, we first would like to recall a related model considered in Klejn and Rozenberg (1980) already in 1980, for the case of L systems, calling them 0*LIP* systems: like in Indian parallel grammars, all identical symbols simultaneously evolve by the *same* rule, but like in Lindenmayer systems, all symbols evolve in parallel. We also note that in the area of P systems such a requirement may be viewed as a special case of the *label agreement* feature. Label selection, target selection, and target agreement have extensively been studied, for example, see Alhazov and Freund (2014, 2015); hence, it is proper to call it *rule agreement*, as studied, e.g., in Alhazov et al. (2015b).

#### **Lemma 7**

*With unconditional halting, pure white hole EESNP systems generate at least* 0*LIP*.

#### *Proof*

Take an arbitrary 0*LIP* system *L* with alphabet $\{A_i\mid 1\le i\le n\}$. We define a pure EESNP system $\varPi$ with *n* neurons as follows. The rules of $\varPi$ consist of one rule

$$\begin{aligned} (i,N_+/all\rightarrow \{(j,all\cdot |w|_{A_j})\mid 1\le j\le n\}) \end{aligned}$$

for every rule $A_i\rightarrow w$ in *L*.

The multiplicity of symbols $A_i$ in a configuration of *L* corresponds to the multiplicity of spikes in neuron *i* of an associated configuration of $\varPi$. Hence, the bisimilarity between derivations in $\varPi$ and derivations in *L* is obvious. $\square$

Clearly, as a particular case with $n=1$, we get the previous example covering *DTU*0*L*.

### Computational completeness of EESNP systems

The following main result was already established in Alhazov et al. (2015a).

#### **Lemma 8**

*The computation of any register machine can be simulated in only one single actor neuron of an EESPN system.*

#### *Proof*

Let $M=\left( n,B,l_0,l_h,P\right)$ be an *n*-register machine, where *n* is the number of registers, *P* is a finite set of instructions injectively labeled with elements from the set of labels *B*, $l_0$ is the initial label, and $l_h$ is the final label.

Then we can effectively construct an EESNP system $\varPi =\left( m,S,R\right)$ simulating the computations of *M* by encoding the contents $n_i$ of each register *i*, $1\le i\le n$, as $p_i^{n_i}$ for different prime numbers $p_i$. Moreover, for each instruction (label) *j* we take a prime number $q_j$, of course, also each of them being different from each other and from the $p_i$.

The instructions are simulated as follows:

- $$l_1:\left( ADD\left( r\right) ,l_2,l_3\right)$$   (ADD instruction)
  This instruction can be simulated by the rules $\left\{ \left( 1,q_{l_1}\cdot {\mathbb{N}}_+/all \rightarrow \left\{ \left( 1,all \cdot q_{l_i}p_{r}/q_{l_1}\right) \right\} \right) \mid 2\le i\le 3 \right\}$ in neuron 1.
- $$l_1:\left( SUB\left( r\right) ,l_2,l_3\right)$$   (SUB instruction)
  This instruction can be simulated by the rules $\left( 1,q_{l_1}p_r\cdot {\mathbb{N}}_+/all \rightarrow \left\{ \left( 1,all \cdot q_{l_2}/\left( q_{l_1}p_r\right) \right) \right\} \right)$ and $\left( 1,\left( q_{l_1}\cdot {\mathbb{N}}_+\setminus q_{l_1}p_r\cdot {\mathbb{N}}_+\right) / all \rightarrow \left\{ \left( 1,all \cdot q_{l_2}/q_{l_1} \right) \right\} \right)$ in neuron 1; the first rule simulates the decrement case, the second one the zero test.
- $$l_h:halt$$   (HALT instruction)
  This instruction can be simulated by the rule $\left( 1,q_{l_h}\cdot {\mathbb{N}}_+/all \rightarrow \left\{ \left( 1,all \cdot 1/q_{l_h}\right) \right\} \right)$ in neuron 1.
  In fact, after the application of the last rule, we end up with $p_1^{m_1}\cdots p_n^{m_n}$ in neuron 1, where $\left( m_1,\ldots ,m_n\right)$ is the vector computed by *M* and now, in the prime number encoding, by $\varPi$ as well.

All the checking sets we use are regular, and the productions in all the white hole rules even again yield integer numbers. $\square$

#### *Remark 2*

As the productions in all the white hole rules of the EESNP system constructed in the preceding proof even again yield integer numbers, we could also interpret this EESNP system as an ESNP system with exhaustive use of rules:

The white hole rules in the EESNP system constructed in the previous proof are of the general form

$$\begin{aligned} \left( 1,q\cdot {\mathbb{N}}_+/all \rightarrow \left\{ \left( 1,all \cdot p/q\right) \right\} \right) \end{aligned}$$

with *p* and *q* being natural numbers. Each of these rules can be simulated in a one-to-one manner by the rule

$$\begin{aligned} \left( 1,q\cdot {\mathbb{N}}_+/q \rightarrow p \right) \end{aligned}$$

used in an ESNP system with one neuron in the exhaustive way.

Based on the preceding main result, i.e., Lemma 8, the following theorems were proved in Alhazov et al. (2015a).

#### **Theorem 6**

*Any recursively enumerable set of* *n*-*dimensional vectors can be generated by an EESNP system with* $n+1$ *neurons.*

#### *Proof*

We only have to show how to extract the results into the additional output neurons from the single actor neuron which can do the whole computational task as exhibited in Lemma 8. Yet this is pretty easy:

When the actor neuron reaches the halting state, the desired result $m_i$ for output neuron $i+1$ is stored as factor in this one number stored in the actor neuron within the prime number encoding, i.e., as ${p_i}^{m_i}$, for $1\le i\le n$. Instead of using the final rule

$$\begin{aligned} \left( 1,q_{l_h}\cdot {\mathbb{N}}_+/all \rightarrow \left\{ \left( 1,all \cdot 1/q_{l_h}\right) \right\} \right) \end{aligned}$$

in neuron 1 we now take the rule

$$\begin{aligned} \left( 1,q_{l_h}\cdot {\mathbb{N}}_+/all \rightarrow \left\{ \left( 1,all \cdot r_1/q_{l_h}\right) \right\} \right) . \end{aligned}$$

With the rules

$$\begin{aligned} \left( 1,r_ip_i \cdot \mathbb{N}_+/all \rightarrow \left\{ \left( 1,all \cdot 1/p_i\right) ,\left( i+1,1\right) \right\} \right) , \end{aligned}$$

we can decode the factor ${p_i}^{m_i}$ to $m_i$ into output neuron $i+1$, with the instruction code (prime number) $r_i$ for $1\le i\le n$. If the contents of the actor neuron is not dividable by $p_i$ any more, we switch to the next instruction code $r_{i+1}$ by the rule

$$\begin{aligned} \left( 1,\left( r_i\cdot {\mathbb{N}}_+ \setminus r_ip_i\cdot {\mathbb{N}}_+\right) /all \rightarrow \left\{ \left( 1,all \cdot r_{i+1}/r_i\right) \right\} \right) . \end{aligned}$$

At the end, we can end up with 0 in the actor neuron after having used the rule

$$\begin{aligned} \left( 1,\left( r_n\cdot {\mathbb{N}}_+ \setminus r_np_n\cdot {\mathbb{N}}_+\right) /all \rightarrow \emptyset \right) \end{aligned}$$

and then stop with $m_i$ in output neuron $i+1$, $1\le i\le n$. $\square$

#### **Theorem 7**

*Any recursively enumerable language* *L* *with* $L\subseteq T^*$ *for a terminal alphabet* *T* *with* $card\left( T\right) =n$ *can be generated by an EESNP system with* $n+1$ *neurons.*

#### *Proof*

In the case of generating strings, we have to simulate a register machine with output tape; hence, in addition to the simulating rules already described in Lemma 8, we have to simulate the tape rule $l_1:\left( write\left( a\right) ,l_2\right)$, which in the EESNPS means sending one spike to the output neuron $N\left( a\right)$ representing the symbol *a*. This task is accomplished by the rule $\left( 1,l_1\cdot {\mathbb{N}}_+/all \rightarrow \left\{ \left( 1,all \cdot l_2/l_1\right) , \left( N\left( a\right) ,1\right) \right\} \right)$. The rest of the construction and of the proof is similar to that what we have done in the proof of Lemma 8. $\square$

## Red–green EESNP systems

For defining a suitable model of red–green EESNP systems we have to consider several constraints.

First of all, the computations should be deterministic, i.e., for any configuration of the EESNP system $\varPi$ there should be at most one rule applicable in each neuron. This condition can be fulfilled syntactically by requiring the checking sets of all the rules in each neuron to be disjoint.

Whereas in the generating case we had one output neuron for each possible input symbol, these specific neurons now have to act as input neurons. As we only want deterministic behavior to be considered now, we assume that in every derivation step at most one input neuron spikes until the whole input is “read”, but this input has to be made “on demand”, i.e., we imagine that the EESNP system $\varPi$ sends out an input request to the environment which is answered in the next step by the spiking of exactly one input neuron as long as the string has not been “read” completely.

“Reading” the spiking of an input neuron into the single actor neuron means that we have to be able to distinguish the signals coming from different input neurons. Hence, the simplest variant to do this is to multiply the spike coming from input neuron number *k* by *k*. Yet then we have to take into account that the minimum value in the actor neuron must be bigger than the maximal number *k*, i.e., the smallest prime number used for the prime number encoding must fulfill this condition, and our encoding of the number $n_i$ now is chosen to be ${p_i}^{n_i+1}$.

Finally, we have to define red and green “states” of the red–green EESNP system; yet as we only have a finite number of rules in each neuron, each of the possible vectors of rules represents a color; hence, the color of the current configuration, i.e., its “state”, can be defined via the (unique) vector of rules to be applied.

Based on the proof Lemma 8, we now can easily establish the following results, similar to the results obtained for red–green register machines, see Lemmas 1 and 2 as well as Theorem 3:

### **Lemma 9**

*The computations of a red–green register machine* *RM* *with an arbitrary number of registers and with string input can be simulated by a red–green EESNP system* $\varPi$ *in such a way that during the simulation of a computation step of* *RM* *leading from an instruction with label (“state”)* *p* *with color* *c* *to an instruction with label (“state”)* $p'$ *with color* $c'$ *the simulating EESNP system* $\varPi$ *uses states of color* *c* *and only in the last step of the simulation changes to a state of color* $c'$.

### **Lemma 10**

*The computations of a red–green EESNP system* $\varPi$ *can be simulated by a red–green register machine* *RM* *with two registers and with string input in such a way that during the simulation of a derivation step of* $\varPi$ *leading from a state* *p* *with color* *c* *to a state* $p'$ *with color* $c'$ *the simulating register machine uses instructions with labels (“states”) of color* *c* *and only in the last step of the simulation changes to a label (“state”) of color* $c'$.

As an immediate consequence, the preceding two lemmas yield the characterization of $\varSigma _2$ and $\varSigma _2\cap \varPi _2$ by red–green EESNP systems:

### **Theorem 8**

(Computational power of red–green EESNP systems)

1. *A set of strings* *L* *is recognized by a red–green EESNP system with one mind change if and only if* $L\in \varSigma _1$, *i.e., if* *L* *is recursively enumerable.*
2. *Red–green EESNP systems recognize exactly the* $\varSigma _2$-*sets of the Arithmetical Hierarchy.*
3. *Red–green EESNP systems accept exactly those sets which simultaneously are* $\varSigma _2$- *and* $\varPi _2$-*sets of the Arithmetical Hierarchy.*

## Conclusion

In this paper, we have further studied the model of extended spiking neural P systems with white hole rules as introduced in Alhazov et al. (2015a). With this variant of extended spiking neural P systems, computational completeness can already be obtained with only one actor neuron, as the computations of any register machine can already be simulated in only one neuron equipped with the most general variant of white hole rules. Using this single actor neuron to also extract the final result of a computation, we even obtain weak universality with only one neuron.

The model of extended spiking neural P systems with white hole rules also allows for a red–green variant and thus to “go beyond Turing”. Computational completeness can already be obtained with only one actor neuron, and with the red–green variant of extended spiking neural P systems with white hole rules exactly the $\varSigma _2$-sets of the Arithmetical Hierarchy can be recognized.

A quite natural feature found in biology and also already used in the area of spiking neural P systems is that of inhibiting neurons or axons between neurons, i.e., certain connections from one neuron to another one can be specified as inhibiting ones—the spikes coming along such inhibiting axons then close the target neuron for a time period given by the sum of all inhibiting spikes, e.g., see Binder et al. (2007). Such variants can also be considered for extended spiking neural P systems with white hole rules.
